# Checkpoint Kinase 1 (Chk1) inhibition fails to activate the Stimulator of Interferon Genes (STING) innate immune signalling in a human coculture cancer system

**DOI:** 10.1186/s43556-021-00044-1

**Published:** 2021-06-20

**Authors:** Teresa Brooks, Joanne Wayne, Andrew J. Massey

**Affiliations:** Vernalis (R&D) Ltd, Granta Park, Abington, Cambridge, CB21 6GB UK

**Keywords:** Chk1, DNA damage, TBK1, STING, Type I interferon response

## Abstract

Utilising Checkpoint Kinase 1 (Chk1) inhibitors to increase cytoplasmic DNA may be a potential strategy to increase the sensitivity of tumours to immune checkpoint modulators. The appearance of DNA in the cytoplasm can drive Cyclic GMP-AMP Synthase-2′,3′-Cyclic Guanosine Monophosphate–Adenosine Monophosphate-Stimulator of Interferon Genes (cGAS-cGAMP-STING) inflammatory, anti-tumour T-cell activity via a type I interferon (IFN) and nuclear factor-κB response. In the THP1-Dual reporter cell line, the STING agonist cGAMP activated both reporters, and increased phosphorylation of the innate immune pathway signallers Tank Binding Kinase 1 (TBK1) and Interferon Regulatory Factor (IRF) 3. Inhibition of Chk1 increased TBK1 but not IRF3 phosphorylation and did not induce IRF or NF-κB reporter activation. cGAMP induced a Type I IFN response in THP1 cells whereas inhibition of Chk1 did not. HT29 or HCC1937 cell treatment with a Chk1 inhibitor increased cytoplasmic dsDNA in treated HCC1937 but not HT29 cells and increased IRF reporter activation in cocultured THP1-Dual cells. HT29 cells pre-treated with gemcitabine or camptothecin had elevated cytoplasmic dsDNA and IRF reporter activation in cocultured THP1-Dual cells. Camptothecin or gemcitabine plus a Chk1 inhibitor increased cytoplasmic dsDNA but Chk1 inhibition suppressed IRF reporter activation in cocultured THP1 cells. In THP1-Dual cells treated with cGAMP, Chk1 inhibition suppressed the activation of the IRF reporter compared to cGAMP alone. These results suggest that, in some cellular models, there is little evidence to support the combination of Chk1 inhibitors with immune checkpoint modulators and, in some combination regimes, may even prove deleterious.

**Supplementary Information:**

The online version contains supplementary material available at 10.1186/s43556-021-00044-1.

## Introduction

Modulators of immune checkpoints, namely antibodies targeting PD-1 (Pembrolizumab, Nivolumab and Cempilimab), PD-L1 (Atezolizumab, Avelumab or Durvalumab) or CTLA4 (Ipilimumab) are at the vanguard of a reinvigorated approach to harness the immune system to kill human cancers [1]. Robust, enduring cures have been achieved with immune checkpoint modulating drugs but often only in a small sub-population of patients. In some cancer types, they have been demonstrated to have no therapeutic benefit. Those tumours that contain increased numbers of tumour infiltrated CD3+ and CD8+ cells (so called “inflamed” or “hot” tumours) tend to exhibit increased responsiveness to T cell checkpoint modulators [2]. Identifying strategies to elevate the responsiveness of so called “cold” tumours to immune checkpoint modulators is currently an important area of research.

Activating the cGAS-STING pathway [1, 3] to increase tumour inflammation thereby turning the tumours from “cold” to “hot” is one such strategy. Cyclic GMP-AMP synthase (cGAS) detects cytoplasmic dsDNA leading to the formation of cyclic GMP-AMP (cGAMP) from GTP and ATP. Binding of cGAMP to STING activates STING leading to an ER to Golgi translocation. Following recruitment and trans-autophosphorylation, TBK1 phosphorylates STING, IRF3, IRF5 and/or IRF7 triggering IFN signalling and gene activation [2]. Typically, cGAS-STING launches an innate immunological response to pathogens such as viruses. Intrartumoral injection of STING agonists induce IFN signalling in the tumour and increase the responsiveness of otherwise refractory tumours to T cell checkpoint therapy such as anti-PD-1 or PD-L1 antibodies. Phase I clinical trials of STING agonists (namely ADU-S100 [4], SB 11285, E7766 and MK-1454), both as single agents and in combination with anti-PD-1, PD-L1 or CTLA4 antibodies are currently underway.

As STING is activated by cytoplasmic dsDNA, therapies that increase cytoplasmic dsDNA may be an alternative strategy for STING activation compared to STING agonist therapy. Increased cytosolic dsDNA induced by DNA damage inducing therapeutic drugs such as radiotherapy, cytotoxic chemotherapy or PARP inhibitors activate the cGAS-STING-IFN response [5–10] with S-phase DNA damage suggested to be a particularly potent activator [11]. The induction of cGAS/STING inflammatory responses following DNA damage by PARP [12] or ATR [13] inhibitors has been demonstrated to induce micronucleation with subsequent leaking of DNA from the micronuclei able to activate the innate immune response [14, 15]. Progression through mitosis of the damaged cells leads to damage induced breaks and their subsequent conversion into micronuclei is suggested to be critical [16]. Blocking cells at the G2-M transition prevents inflammation-stimulated gene expression following damage [17]. Conversely, cells with DNA damage must complete mitosis as during mitosis, cGAS/STING activity is suppressed to prevent self-DNA recognition [18].

Chk1 is an important transducer of the DNA damage response pathway, a key pathway responsible for protecting cells from DNA damage induced by endogenous and exogenous sources [19, 20]. Chk1 inhibitors induce replication stress and S-phase DNA damage in tumour but not normal cells. Underlying DNA repair defects or high levels of replication stress often make tumour cells hypersensitive to Chk1 inhibition. Inhibitors of Chk1 (prexasertib, SRA737) are currently being evaluated as potential cancer therapeutics either as monotherapy or in combination with standard of care chemotherapy drugs and ionizing radiation [21, 22].

We have previously demonstrated the failure of Chk1 inhibitors to induce a Type I IFN response in solid tumour cell lines growing in culture [23]. Here we further expand these studies to leukaemia derived cell lines, and subsequently in a coculture system with pre-treated human solid cancer cell lines. As observed previously, Chk1 inhibition again failed to induce a Type I IFN response in either of these cell model systems. These results therefore provide important insights when planning clinical trials combining Chk1 inhibitors and immune checkpoint modulators.

## Results

### DNA damage response inhibition does not induce a type I interferon response in THP1 or Jurkat cancer cells

**Fig. 1:**
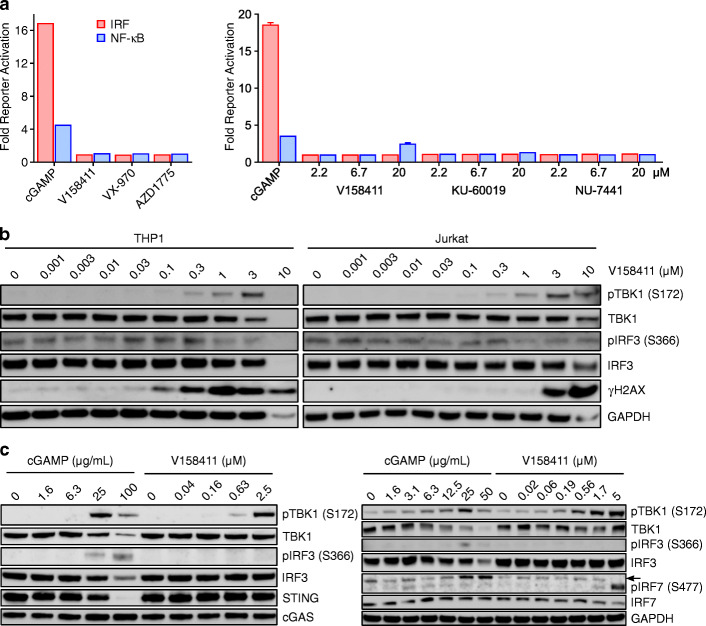
DNA damage response inhibitors do not increase IFN or NF-kB reporter activation in THP1-Dual cells. **a** THP1-Dual cells were treated with 12.5 μg/mL cGAMP, 2.5 μM V158411 (Chk1i), VX-970 (ATRi), AZD1775 (Wee1i) or 2.2–20 μM KU-60019 (ATMi) or NU-7441 (DNA-PKcsi) for 24 h and reporter activity determined as described. Mean of 2 independent wells. **b** THP1 or Jurkat cells were treated with the indicated concentrations of V158411 for 24 h. **c** THP1-Dual cells were treated with the indicated concentrations of cGAMP or V158411 for 24 h

Proliferation of acute monocytic leukaemia THP1 and acute T cell leukaemia Jurkat cells (Supplementary Table 1) was robustly inhibited by the potent Chk1 inhibitor V158411 [24]. THP1 cells have been routinely used to study the cGAS / STING pathway as they induce a Type I IFN response to cGAMP and dsDNA that is cGAS / STING dependent [25, 26]. In comparison, Jurkat cells express approximately 1000-fold less cGAS mRNA than THP1 cells and no detectable cGAS protein, and dsDNA cannot activate cGAS-STING signalling [27]. γH2AX, a marker of DNA double-strand breaks and replication stress, is increased in a host of cell lines including leukaemia and lymphoma cells [28] following Chk1 inhibition. Increased DNA damage has been demonstrated to activate the innate immune response and NF-κB signalling pathways. The THP1-Dual reporter cells stably express both a secreted *Lucia* luciferase and an embryonic alkaline phosphatase reporter gene. The luciferase is controlled by five IFN-stimulated response elements whilst the alkaline phosphatase by three c-Rel binding sites and five NF-κB consensus transcriptional response elements. The IRF and NF-κB reporters in this cell line are robustly activated by a range of ligands including cGAMP, poly(I:C) and poly (dA:dT). The ability of DNA damage response inhibitors to activate the IRF or NF-κB reporter in the THP1-Dual cells was evaluated. Treatment of THP1-Dual cells with the cyclic dinucleotide cGAMP robustly activated both the IRF and NF-κB reporters by approximately 16- and 4-fold respectively (Fig. 1a). IRF reporter activation occurred within 6 h following cGAMP treatment reaching a maximum at 24 h and then slowly declining over the next 48 h (Supplementary Fig. 1a). NF-κB reporter activation was more delayed with activity not detected until 24 h after cGAMP addition but remaining stable out to at least 72 h. In comparison, treatment with inhibitors of Chk1 (V158411), Ataxia Telangiectasia and Rad3-Related Protein (ATR, VX-970) [29], WEE1 G2 Checkpoint Kinase (Wee1, AZD1775) [30], Ataxia Telangiectasia Mutated Protein Kinase (ATM, KU-60019) [31] or DNA-dependent Protein Kinase, catalytic subunit (DNA-PKcs, NU7441) [32] failed to activate either the IRF or NF-κB reporters. This appeared time independent as a shorter (6 h) or longer (48 or 72 h) treatment did not activate expression of either reporter. At the longer time points of 48 and 72 h, V158411 appeared to decrease reporter expression below that of the basal level. In THP1 and Jurkat cells, V158411 concentration-dependently increased TBK1 phosphorylation on S172 approximately in line with increased γH2AX (Fig. 1b). Increased phosphorylation of TBK1 did not lead to increased activation of IRF3 through phosphorylation on S366. TBK1 phosphorylation appeared to occur rapidly within 3 h of V158411 addition and remained sustained up to at least 48 h (Supplementary Fig. 1b). In contrast, treatment of THP1 cells with cGAMP increased TBK1 phosphorylation as well as phosphorylation of IRF3 and IRF7 (Fig. 1c).

**Fig. 2:**
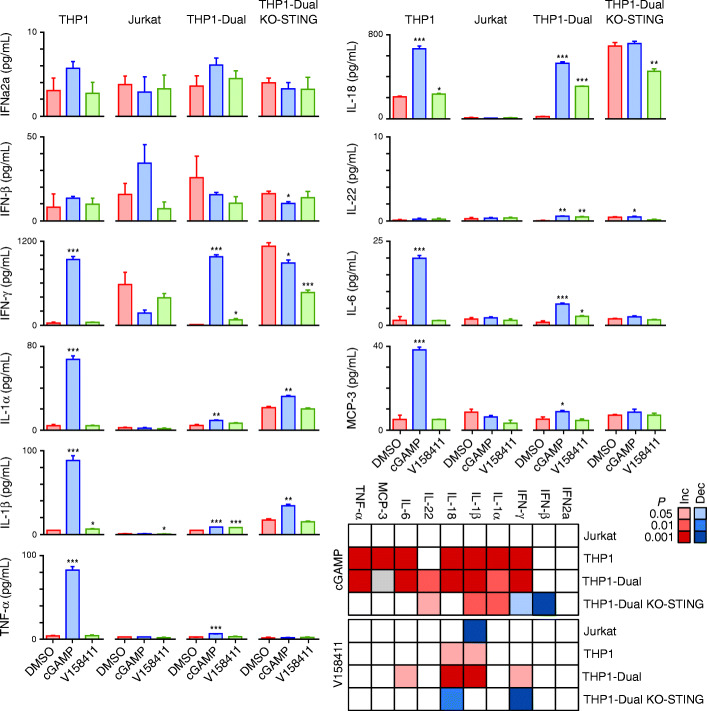
Chk1i induced cytokine changes in haematological cancer cell lines. Cells were treated with V158411 or cGAMP for 24 h. Cytokines from treated cell lysates were detected on a U-plex 10 spot ELISA. Mean of 3 independent determinations ± SD

A multiplex cytokine ELISA assay was utilised to determine whether Chk1 inhibition induced a type I interferon response in THP1 or Jurkat cells following 24-h treatment. cGAMP increased IFNγ, IL-1α, IL-1β, IL-18, IL-6, MCP and TNFα in the THP1 and THP1-Dual but not the Jurkat cells (Fig. 2). This appeared to be STING dependent as the response to cGAMP in the THP1-Dual KO-STING cell line (generated from THP1-Dual cells by stable knockout of the STING gene) were attenuated compared to the parental THP1-Dual cells. Surprisingly, significant differences in cytokine production following cGAMP treatment between the original THP1 cells (obtained from the ATCC) and the reporter containing THP1-Dual cells was noted with the THP1-Dual cells generally producing significantly reduced total cytokines than the ATCC THP1 cells. In comparison, V158411 induced very little changes to the cytokine levels with IL-1β and IL-18 increased in the THP1 and THP1-Dual cells as well as IFN-γ and IL-6 in the THP1-Dual cells. These changes in the THP1-Dual cells appeared to be STING dependent as STING KO attenuated these (Fig. 2).

### V158411 increases cytoplasmic dsDNA in human cancer cell lines

**Fig. 3:**
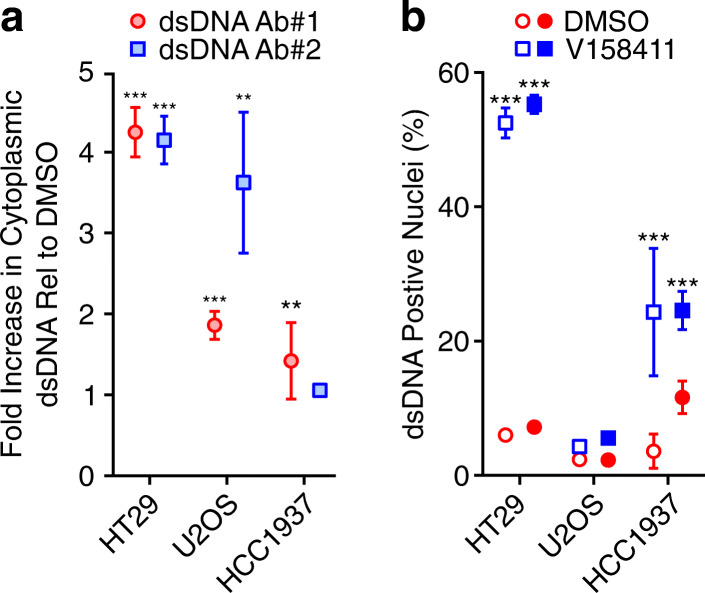
V158411 increases cytoplasmic dsDNA in cancer cell lines. **a** Cells were treated with 3x γH2AX EC_50_ V158411 (2.4, 1.9 and 0.78 μM for HT29, U2OS and HCC1937 respectively) for 24 h and cytoplasmic dsDNA determined by high content imaging with two different mouse monoclonal antibodies. **b** Nuclei staining positive for dsDNA were determined from (**a** and **b** adapted from [23]). Open and filled symbols indicate data from the two different mAbs. Mean of 3 independent wells ± SD

Chk1 inhibitors, including V158411, increase DNA damage and replication stress, as measured by increased pan-nuclear γH2AX, in HT29, U2OS or HCC1937 cells with EC_50_ values (γH2AX EC_50_) of 0.80, 0.64 and 0.26 μM respectively [23, 33] (Supplementary Table 1). Increased DNA damage can result in increased cytoplasmic DNA. Two different anti-dsDNA mouse monoclonal antibodies coupled with high content imaging were utilised to measure cytoplasmic dsDNA. 3-times the γH2AX EC_50_ (3x γH2AX EC_50_) of V158411 for 24 h increased cytoplasmic dsDNA in HT29 cells 4–5-fold compared to DMSO treated control cells (Fig. 3a, adapted from [23]). In U2OS cells, the increase in cytoplasmic dsDNA by V158411 was less robust being 2–4-fold dependent on the mouse monoclonal antibody used. In comparison, V158411 did not dramatically alter dsDNA levels in HCC1937 cells with a maximal increase in cytoplasmic dsDNA of around 1.5-fold. This method of determining cytoplasmic dsDNA staining by high content imaging requires saponin permeabilisation. This does not permeabilise the nuclear membrane. After DMSO treatment, < 10% of cells had nuclear dsDNA staining. Twenty-four hours treatment with V158411, however, significantly increased the fraction of HT29 and HCC1937 but not U2OS nuclei staining anti-dsDNA antibody positive (Fig. 3b, adapted from [23]). This suggests that V158411 treatment of HT29 and HCC1937 cells resulted in reduced nuclear membrane integrity.

### Chk1 inhibitor pre-treatment of cancer cells increases IRF-dependent reporter activation in cocultured THP1-Dual reporter cells

**Fig. 4:**
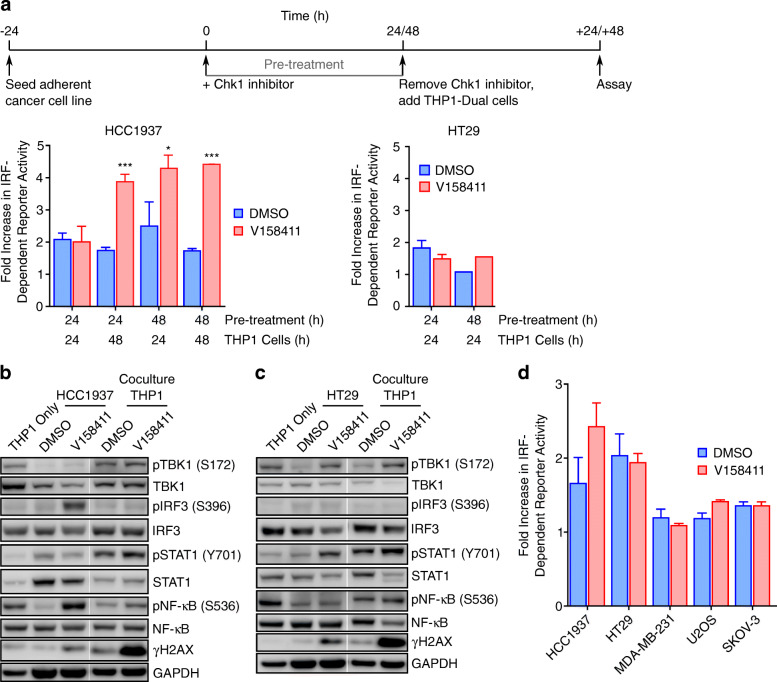
Cancer cells pre-treated with V158411 increased IRF-dependent reporter activation in cocultured THP1-Dual reporter cells. **a** THP1 cells were cocultured with V158411 pre-treated HT29 or HCC1937 cells and IRF-dependent reporter activation determined. Mean of 3 independent determinations ± SD. **b** HCC1937 or **c** HT29 cells were pre-treated with V158411 and then cocultured with THP1-Dual cells. Protein lysates were analysed by western blotting. **d** THP1 cells were cocultured with V158411 pre-treated adherent cancer cell lines and IRF-dependent reporter activation determined. Mean of 3 independent determinations ± SD

The innate immune response has been demonstrated to be activated by cytoplasmic dsDNA resulting in a robust type I interferon response [34]. We have previously demonstrated that Chk1 inhibition in solid cancer cell lines such as HCC1937 or HT29 failed to activate the cGAS/STING pathway downstream of TBK1 [23]. However, damage-associated molecular patterns (DAMPs) (endogenous danger molecules release for damaged or dying cells) can activate components of the innate immune system such as natural killer cells, dendritic cells, or monocytes/macrophages [35, 36]. We therefore utilised a coculture system composed of Chk1 inhibitor pre-treated adherent cancer cell lines along with the monocytic THP1 cell line (THP1-Dual) harbouring an IRF reporter as the sensor. V158411 pre-treated HCC1937 or HT29 cells were subsequently cocultured with the THP1-Dual reporter cells and the IRF-dependent reporter activity determined. The experiments were designed such that THP1-Dual cells were never directly exposed to V158411 (Fig. 4a). V158411 pre-treated HCC1937 cells increased reporter activation in the THP1-Dual cells between 4 and 5-fold whilst V158411 pre-treated HT29 cells did not. This increase in IRF-dependent reporter activation did not appear to correlate with increases in nuclear γH2AX induced by V158411 in the pre-treated cancer cells (Supplementary Fig. 2).

The effect of V158411 on IRF pathway activation in the coculture system was further evaluated by western blotting. Treatment of HCC1937 but not HT29 cells with V158411 appeared to increase both IRF3 and NF-κB phosphorylation but not Signal Transducer and Activator of Transcription Protein 1 (STAT1) phosphorylation (Fig. 4b and c). This increase in pIRF3 and pNF-κB was, however, not apparent in the THP1 cells cocultured with the treated HCC1937 cells (Fig. 4b). Likewise, co-culture of THP1 cells with V158411-treated HT29 cells did not increase IRF3, NF-κB or STAT1 phosphorylation in these cells (Fig. 4c). We further evaluated the effect of V158411 pre-treatment on IRF-dependent reporter activation in cocultured THP1 cells in several additional cell lines. In MDA-MB-231, SKOV3 or U2OS cells pre-treated with V158411, no increase in IRF-reporter activation in the co-cultured THP1-Dual cells was observed (Fig. 4d).

### Chk1 inhibition blocked IRF-dependent reporter activation in THP1-Dual reporter cells cocultured with HT29 cells pre-treated with cytotoxic chemotherapy

**Fig. 5:**
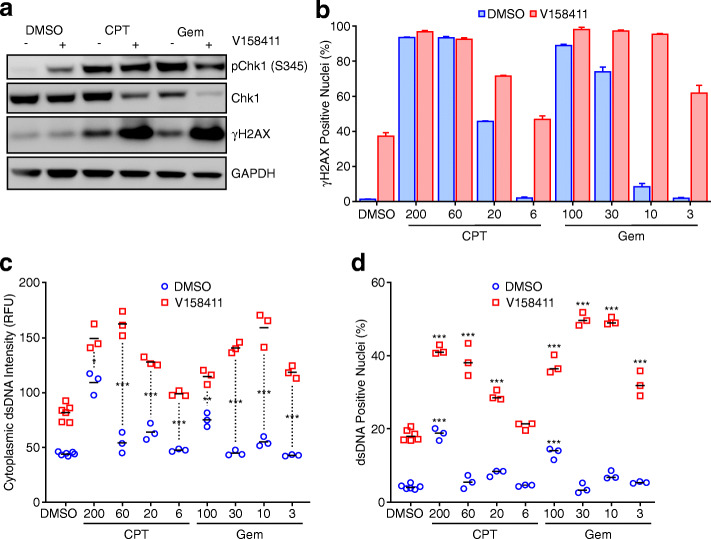
Cytoplasmic dsDNA was increased in cells sequentially treated with camptothecin or gemcitabine then V158411. **a** HT29 cells were treated with 100 nM camptothecin (CPT) or 50 nM gemcitabine (Gem) for 18 h followed by 0.3 μM V158411 for a further 24 h. **b** HT29 cells were treated with camptothecin (CPT) or gemcitabine (Gem) for 18 h followed by 0.3 μM V158411 for a further 24 h. **c** HT29 cells were treated with 6–200 nM camptothecin (CPT) or 3–100 nM (Gem) gemcitabine for 18 h followed by 0 or 0.3 μM V158411 for a further 24 h. Anti-dsDNA staining was determined by high content imaging (Adapted from [23]). **d** dsDNA positive nuclei were determined from **c**. 3 independent determinations; horizontal bar, mean

The topoisomerase inhibitor camptothecin and the ribonucleotide reductase inhibitor gemcitabine are routinely used cancer chemotherapeutics that increase DNA damage (as measured by an increase in nuclear γH2AX and pChk1 (S345)) (Fig. 5a and b). Chk1 inhibition increased DNA damage induced by both agents leading to increased cytotoxicity [37, 38]. Cytoplasmic dsDNA can be increased following chemotherapy induced DNA damage. Cytoplasmic dsDNA staining was increased in HT29 cells treated with 200 nM camptothecin or 100 nM gemcitabine (Fig. 5c, adapted from [23]). In addition, the fraction of cells staining positive for nuclear dsDNA (Fig. 5d) under these permeabilisation conditions increased indicative of a disrupted nuclear membrane. Cytoplasmic dsDNA as well as the fraction of cells with a disrupted nuclear membrane was increased following camptothecin or gemcitabine treatment in combination with V158411 compared to chemotherapy drug alone. Lower concentrations of camptothecin or gemcitabine (20 nM and 3 nM respectively) increased dsDNA when they were combined with V158411.

**Fig. 6:**
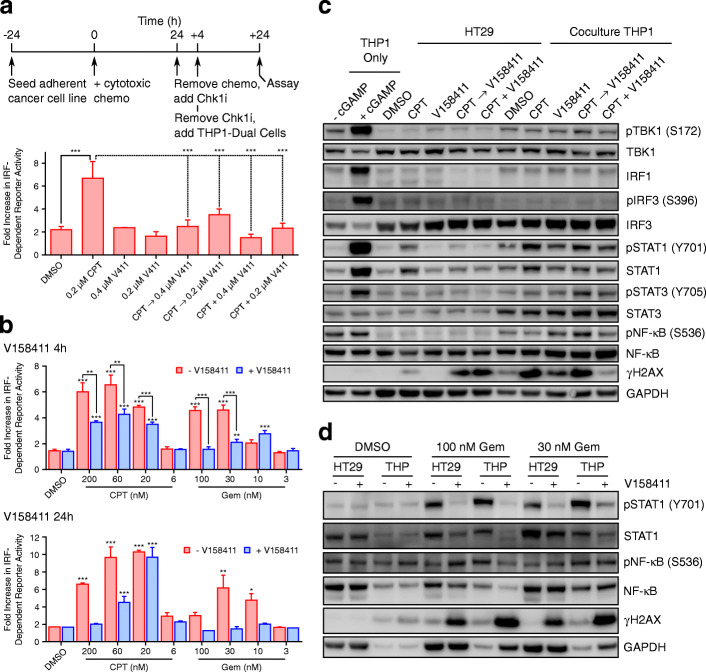
V158411 inhibits IRF-reporter activation in THP1-Dual cells cocultured with camptothecin or gemcitabine pre-treated HT29 cells. **a** IRF-dependent reporter activity was determined in THP1 cells cocultured with camptothecin (CPT) plus V158411 pre-treated HT29 cells. Mean of 3 independent determinations ± SD. **b** IRF-dependent reporter activity was determined in THP1 cells cocultured with HT29 cells pre-treated with camptothecin (CPT) or gemcitabine (Gem) plus V158411. Mean of 3 independent determinations ± SD. **c** THP1 cells were cocultured with HT29 cells pre-treated with 200 nM camptothecin (CPT) plus V158411 for 24 h. **d** THP-1 cells were cocultured with HT29 cells pre-treated with gemcitabine (Gem) plus V158411. Lysates were analysed by western blotting

We utilised the same coculture system described previously to evaluate the effects of cytotoxic chemotherapy in combination with a Chk1 inhibitor on the THP1-Dual IRF-driven reporter cell line. HT29 cells were pre-treated with camptothecin for 18 h, followed by V158411 for 4 h before being cocultured with THP1-Dual cells for a further 24 h such that the THP1 cells were never directly exposed to either camptothecin or V158411 (Fig. 6a). Camptothecin pre-treatment of HT29 cells resulted in a 7-fold induction of IRF-dependent reporter activation in the cocultured THP1 cells (Fig. 6b). V158411 blocked the camptothecin-induced IRF-dependent reporter activation in the THP1 cells to almost control levels. Likewise, simultaneous treatment with camptothecin plus V158411 resulted in no subsequent reporter activation by camptothecin.

Camptothecin or gemcitabine increased IRF-reporter activation in a concentration-dependent manner (Fig. 6b). V158411 attenuated the reporter activation by camptothecin or gemcitabine, with the effects especially marked in gemcitabine pre-treated HT29 cells. A shorter (4 h) exposure of the HT29 cells to V158411 prior to coculture with the THP1 cells appeared more effective at suppressing the IRF-dependent reporter activation by camptothecin than a longer incubation of 24 h.

The consequences of the combination treatment on IRF pathway signalling were evaluated by western blotting. Camptothecin treatment increased STAT1 phosphorylation in both the treated HT29 cells and cocultured THP1 cells which was attenuated by V158411 (Fig. 6c). In combination with gemcitabine, this effect was even more marked. Gemcitabine robustly increased STAT1 phosphorylation in the HT29 cells and the cocultured THP1 cells. V158411 treatment after gemcitabine effectively inhibited this increase in STAT1 phosphorylation in HT29 cells, reducing pSTAT1 back to control levels (Fig. 6d) in both cell lines.

### Chk1 inhibition blocks IRF-reporter activation by cGAMP

**Fig. 7:**
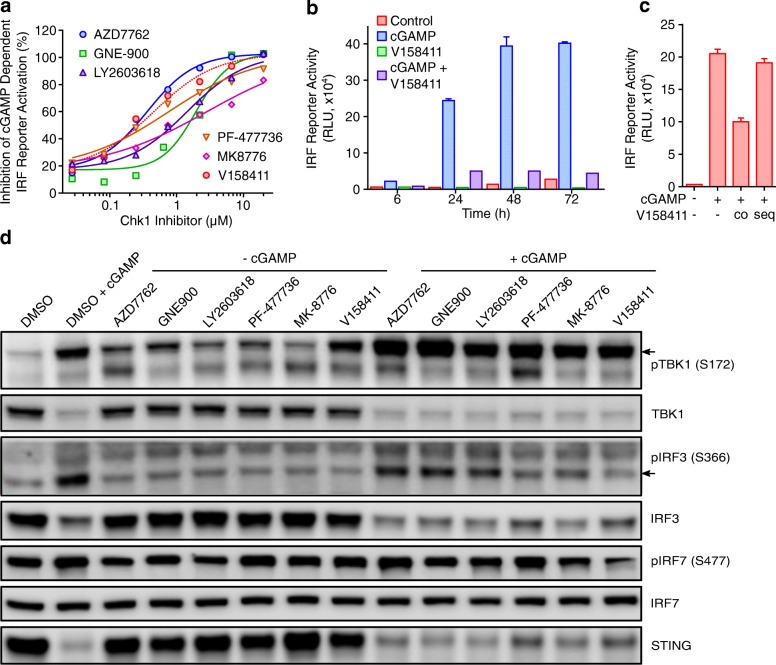
Chk1 inhibitors decrease IRF reporter activity in THP1-Dual-WT cells stimulated with cGAMP. **a** THP1-Dual cells were treated with 50 μg/mL cGAMP plus the indicted concentration of Chk1i for 24 h. **b** THP1-Dual cells were treated with 50 μg/mL cGAMP ±3 μM V158411 for 6–72 h. **c** THP1-Dual cells were treated with 50 μg/mL cGAMP ±1 μM V158411 simultaneously for 24 h (co) or with cGAMP for 24 h then V158411 for 30 min (seq). Mean of 3 independent wells ± SD. **d** THP1-Dual cells were treated with the IC_80_ value of Chk1i determined in **a** above in combination with 0 or 50 μg/mL cGAMP for 24 h

Given some of the results observed, we evaluated whether Chk1 inhibition may be inhibiting (as well as potentially activating), the IRF dependent responses to Chk1i induced DNA damage. cGAMP is a well described activator of the cGAS/STING/IRF signalling axis [39] and we therefore subsequently evaluated the effect of Chk1 inhibition on the cGAMP driven activation of the IRF reporter construct in the THP1-Dual cells. V158411 inhibited cGAMP-induced reporter activation in the THP1-Dual cells with an IC_50_ of 0.17 μM (Fig. 7a). This effect was apparent across a range of structurally diverse Chk1 inhibitors with IC_50_s in the range 0.4–2.8 μM. This inhibition of cGAMP-induced IRF reporter activation by V158411 was evident 72 h post compound addition (Fig. 7b). To confirm that the effect was not due to compound inhibition of the *Lucia* luciferase protein, no inhibition of the IRF reporter was observed when THP1-Dual cells were stimulated with cGAMP for 24 h then V158411 for 30 min (Fig. 7c). This inhibition of the IRF pathway appeared to occur downstream of STING and TBK1 as cGAMP induced TBK1 phosphorylation and STING protein degradation occurred in the absence or presence of a Chk1 inhibitor (Fig. 7d). The effects on IRF3 phosphorylation were less clear with some inhibition of IRF3 pS366 apparent in cells treated with cGAMP plus Chk1 inhibitor compared to those treated with cGAMP alone.

## Discussion

Modulators of the immune checkpoints, in particular antibodies inhibiting CTLA4 (Ipilimumab), PD-1 (Cempilimab, Nivolumab or Pembrolizumab) or PD-L1 (Durvalumab, Avelumab or Atezolizumab) have heralded a new dawn for cancer therapy. Targeting these checkpoints removes the brakes from the immune system allowing an immune response against the tumour to be mounted [40]. Cures induced by these drugs can be robust but only a small subset of patients appear to respond with some cancer types appearing completely refractory to this immunotherapy. These therapies appear to work best against tumours described as “hot” where the tumour is inflamed with many infiltrated T-cells [41, 42]. Methods to increase the inflamed nature of tumours with agonists of STING (for example ADU-S100 [4] and MK-1454) is one such potential approach with Phase I trials [1, 3] currently underway to investigate this. One drawback of these STING agonists is that direct intratumoural injection is required to negate auto-immune responses. cGAS-STING senses cytoplasmic DNA and triggers an inflammatory response [2]. We, therefore, hypothesised that it could be possible to induce a tumour specific STING response through Chk1 inhibitor induced increases in tumour-cell specific DNA damage and cytoplasmic dsDNA.

Previously, we observed that Chk1 inhibition increases cytoplasmic dsDNA in a diverse range of human cancer cell lines. Increased TBK1 phosphorylation but not activation of any of the downstream pathways from this such as increased pIRF3 or pIRF7, pNF-κB or increased Type I IFNs [23] was observed. Here, we evaluated the effects of Chk1 inhibition in the monocytic leukaemia cell line THP1 harbouring reporters of IFN and NF-κB that respond robustly to the STING agonist cGAMP. In this cell line, Chk1i induced DNA damage but did not induce a type I interferon response. Robust activation of TBK1 was observed but this did not translate into downstream activation of IRF gene transcription and IL-1/TNF-α production. In fact, Chk1i treatment appeared to block cGAMP induced activation of IRF. When these THP1 cells were co-cultured with Chk1i pre-treated solid cancer cell lines, a small increase in IRF-dependent reporter activation was observed. Similar observations were made when the THP1 cells were cocultured with DNA damaging agent-treated cancer cells. Coculture of THP1 cells with camptothecin or gemcitabine pre-treated HT29 colon cancer cells increased IRF-dependent reporter activation in the THP1 cells. However, when the HT29 cells were pre-treated with camptothecin or gemcitabine followed by a Chk1i, IRF reporter activation was supressed despite increased dsDNA observed in the combination treated HT29 cells.

These results strongly suggest that Chk1 inhibition, whilst having the potential to increase DNA damage and cytoplasmic dsDNA, may also have the potential to suppress the pathway as well. Gemcitabine and camptothecin increased STAT1 phosphorylation on Y701 in both the HT29 and THP1 cells which was effectively suppressed by V158411. STAT1 is activated by a range of stimulants including TNF-α and IFN-γ leading to Y701 phosphorylation by the Jak kinases. How Chk1 is interfering with signalling to STAT1 is still unclear.

A recently published paper suggests an important role for Chk1 in phosphorylation of IRF3 on S173 and S175 [43] with inhibition of Chk1 with the Chk1i MK-8776 (SCH 900776) inhibiting Type I IFN mRNA upregulation following either etoposide or camptothecin treatment. In this manuscript, we demonstrate that Chk1 inhibition is also capable of blocking IRF reporter activation in response to cGAMP further supporting a role for Chk1 kinase activity in IRF activation. However, as the cocultured THP1 cells in the combination experiments never received any Chk1 inhibitor treatment, how might Chk1 inhibition be occurring? We have recently demonstrated that Chk1 inhibitors can induce a DNA damage bystander effect in an identical coculture system [44]. When THP1 cells were cocultured with HT29 cells pre-treated with camptothecin and a Chk1i, increased γH2AX and pChk1 (S345), as well as decreased pChk1 (S296) was observed, despite the THP1 cells never being directly exposed to the Chk1i. The decrease in pChk1 (S296), the site of Chk1 autophosphorylation, indicates that Chk1 kinase activity is inhibited in the THP1 cells and therefore potentially negatively regulating the activation of IRF by camptothecin.

This current work is in direct contrast to previously published work demonstrating that Chk1 inhibition in small cell lung cancer cells increased TBK1 and IRF3 phosphorylation, CCL5, IFN-β and CXCL10 mRNA expression, and elevated PD-L1 expression all indicative of a type I IFN response [45]. In a similar study, the Chk1 inhibitor SRA737 in combination with low dose gemcitabine increased CCL5, IFN-β and CXCL10 mRNA expression in small cell lung cancer [46] again indicative of Chk1i activating a Type I IFN response. Finally, a recent paper by Chen and colleagues [17] demonstrated that cell cycle checkpoints can cooperate to suppress DNA- and RNA-associated molecular pattern recognition and anti-tumour immune responses. In this paper, they demonstrate that prolonged G2 arrest induced by either high levels of DNA damage or CDK1 inhibition inhibits inflammation-dependent gene expression in response to this damage. Abrogation of the G1/S and G2/M checkpoints through ATR inhibition and p53 loss, in combination with ionising radiation (IR), upregulated inflammatory signalling that was dependent on RIG-I rather than cGAS. The two main cell lines used in Chen et al (MCF10A and RPE-1) are pseudo normal cells (rather than cancer derived) and were damaged with IR. These cell lines and DNA damaging agent are significantly different to those utilised in this manuscript and point to inflammatory responses to drug-induced DNA damage being cell line and DNA damaging agent dependent.

The studies and data presented in here exhibit some initially apparent inconsistencies and contradictions. For example, in HT29 cells Chk1 inhibition increased greater levels of cytoplasmic dsDNA than in HCC1937 cells but, paradoxically in the coculture system, it is THP1 cells cocultured with Chk1i treated HCC1937 and not HT29 cells where activation of the IRF reporter system is observed. Activation of the IRF signalling is not limited to cytoplasmic DNA and the cGAS/STING pathway. Numerous additional factors including (but not limited to) dsRNA, DAMPs [36, 47] and pathogen-associated molecular patterns (PAMPs [48, 49]) can activate IRF signalling through a host of additional pathways. Therefore, it is conceivable that the signal from the Chk1i treated HT29 cells activating the IRF reporter in cocultured THP1 cells is distinct to dsDNA and the same signal is not generated in Chk1i treated HCC1937 cells. Further work is needed to understand what additional DAMPs beyond cytoplasmic DNA, are generated in Chk1i treated cancer cells.

In this study, we observed that Chk1 inhibitor induced DNA damage failed to activate an innate immune response despite increased cytoplasmic dsDNA and could even suppress the induction following cGAMP or genotoxic drug treatment. This was in direct contrast to previously published studies, in different cell models, where Chk1 inhibitors did induce an innate immune response. These results suggest that, in some cellular models and systems, the clinical use of the combination of Chk1 inhibitors with immune checkpoint modulators is not supported by our data and, in some combination regimes, may even prove deleterious. Further work is needed to understand the differences in innate immune responses to DNA damage in different cell lines and types to aid the clinical development of these combination regimens.

## Materials and methods

### Cell lines and cell culture

HT29, HCC1937, U2OS, Jurkat or THP1 cells were purchased from the American Type Culture Collection (ATCC, LGC Standards, Teddington, UK) and THP1-Dual and THP1-Dual KO-STING cells from Invivogen (thpd-nfs and thpd-kostg, Toulouse, France). HT29 and U2OS cells were cultured in DMEM, and HCC1937, Jurkat and THP1 in RPMI all containing 10% FCS and 1% penicillin/streptomycin (complete media) at 37 °C, 5% CO_2_ in a humidified atmosphere. THP1-Dual and THP1-Dual KO-STING cells were maintained in RPMI containing 10% FCS, 1% penicillin/streptomycin, 0.2% normicin, 200 μg/mL Zeocin and 20 μg/mL Blasticidin. For all coculture experiments, the THP1-Dual cells were grown in the absence of Zeocin / Blasticidin for at least 72 h prior to coculture with other cancer cell lines.

### Co-culture experiments

For reporter assays: 2 × 10^4^ HT29 cells were plated per well of a 96 well plate and allowed to attach overnight. Cells were treated with 3-times the γH2AX EC_50_ of V158411 for 24 h. Media was removed and replaced with 200 μL THP1-Dual cells (in Zeocin / Blastacidin free media) at 2 × 10^5^ cells/mL. After a further 24-h incubation, IRF reporter activity was determined using coelenterazine (3 μM final concentration, Santa Cruz Biotechnology, Dallas, TX) and read on a Victor plate reader (Perkin Elmer, Sear Green, UK) after a 10-min incubation at room temperature. For the combination treatment experiments, HT29 cells were treated with camptothecin or gemcitabine for 18 h then 0.4 μM V158411 for a further 4 h before media was removed and replaced with THP1 cells as above.

For western blot: HT29 or HCC1937 cells (3 mL at 1.67 × 10^5^ cells/mL) were plated per well of a 6 well plate. After being allowed to adhere overnight, cells were treated with 3-times the γH2AX EC_50_ of V158411 for either 24 (HT29) or 48 h (HCC1937). The media was removed and replaced with 3 mL of THP1 cells at 3.33 × 10^5^ cells/mL. After a further 24 or 48 h, the THP1 cells were aspirated and collected by centrifugation. Separate cell lysates were prepared for the adherent and suspension cell lines. For the combination treatment experiments, the HT29 or HCC1937 cells were plated as above and treated with camptothecin or gemcitabine for 24 h followed by 0.4 μM V158411 for a further 4 h. The media was removed and replaced with THP1 cells. After a further 24-h incubation, cells were harvested and lysates prepared.

### Compounds

V158411 was from Vernalis R&D. LY2603618, MK-8776, AZD7762, PF-477736, VX-970, AZD1775, KU-60019 and NU-7441 were purchased from Selleckchem (Houston, TX), and GNE-900 was synthesized in house according to the literature. Stock solutions at 20 mM were prepared in DMSO. Gemcitabine (Apin Chemicals Ltd., Oxford, UK) was prepared as a 20 mM stock in H_2_O and camptothecin (LC Laboratories, Woburn, MA) as a 5 mM stock in DMSO. cGAMP was purchased from Invivogen.

### Antibodies

Supplementary Table 2 lists all the antibodies, and the appropriate dilutions, used in this paper.

### Immunoblotting

After washing once with PBS, cells were lysed in RIPA buffer containing protease and phosphatase inhibitor cocktails (Thermo Fisher Scientific, Hemel Hempstead, UK). Protein concentration was determined using a BCA kit (Thermo Fisher). SDS-PAGE was used to separate proteins in lysates and the antibodies listed in Supplementary Table 2 used for immunoblot analysis. Densitometric analysis was conducted using Image J software (NIH).

### Single cell immunofluorescent imaging

Seven thousand five hundred cells were plated per well of a CellCarrier Ultra 96 well plate (Perkin Elmer) and allowed to adhere overnight. Cells were treated with compounds and then fixed in 4% formaldehyde in PBS at room temperature for 15 min. Cells were blocked and permeabilised with 5% normal goat serum, 0.3% Triton X100 in PBS at room temperature for 60 min. Plates were incubated with primary antibodies (see Supplementary Table 2 for details) diluted in 1% BSA, 0.3% Triton X100 in PBS for 18 h at 4 °C. After PBS washing, plates were incubated with anti-rabbit or anti-mouse Alexa488 conjugated secondary antibodies (Thermo Fisher) diluted in 1% BSA, 0.3% Triton X100 in PBS containing Hoechst 33342 for 45 min at room temperature. After PBS washing, plates were imaged using an Operetta high content imager (Perkin Elmer) equipped with a 10x or 20x objective. Fluorescence intensity of various cellular compartments was determined using Harmony software (Perkin Elmer).

For determination of cytoplasmic dsDNA content, cells were permeabilised with 0.02% saponin for 5 min at room temperature prior to blocking with 5% normal goat serum in PBS. Antibody detection was performed as above except Triton X100 was omitted from all buffers.

### Multiplex cytokine assay

1.5 × 10^6^ Jurkat, THP1 (ATCC), THP1-Dual or THP1-Dual STING KO cells were treated with 3x GI_50_ V158411 or 50 μg/mL cGAMP for 24 h. Cells were harvested by centrifugation and lysed in 100 μL ice cold lysis buffer on ice for 30 min. Cell debris was pelleted and 25 μL of the supernatant added to a 10 spot U-PLEX biomarker plate pre-conjugated with anti-TNF-α, MCP-3, IL-6, IL-22, IL-18, IL-1β, IL-1α, IFN-γ, IFN-β and IFN2a capture antibodies (MesoScale Discovery, Rockville, MD). The plate was developed according to the manufacturer’s instructions and read using a SECTOR Imager 2400.

### Statistical analysis

GraphPad Prism software (version 7.04, GraphPad Software, La Jolla, CA) was utilised for statistical analysis with the following tests: t test for two-way comparisons or a one-way ANOVA with Dunnett’s post-hoc analysis for multiple comparisons.

## Supplementary Information

**Additional file 1: Supplementary Table 1.** GI_50_ and γH2AX EC_50_ values for V158411. **Supplementary Table 2.** List of antibodies used. **Supplementary Fig. 1.** cGAMP but not V158411 increases IFN or NF-kB reporter activation in a time dependent fashion. THP1-Dual cells were treated with 50 μg/mL cGAMP or 3 μM V158411 for 6-72 hours and a reporter activity determined, or b samples prepared for western blotting. Mean of 3 independent wells ± SD. **Supplementary Fig. 2.** V158411 induces γH2AX in HCC1937 or HT29 cells. a HCC1937 or b HT29 cells were treated with 3x GI_50_ of V158411 for 24 or 48 hours. After the THP1-Dual reporter activity was determined, the THP1 cells were removed and the HCC1937 or HT29 cells formaldehyde fixed and γH2AX expression determined by high content imaging. Mean of 3 independent determinations ± SD.

## Data Availability

All available upon request.

## Abbreviations

ATM: Ataxia Telangiectasia Mutated Protein Kinase
ATR: Ataxia Telangiectasia and Rad3-Related Protein
cGAMP: 2′,3′- Cyclic Guanosine Monophosphate–adenosine Monophosphate
cGAS: Cyclic GMP-AMP Synthase
Chk1: Checkpoint Kinase 1
DAMP: Damage-associated molecular pattern
DNA-PKcs: DNA-dependent Protein Kinase, catalytic subunit
IFN: Interferon
IRF: Interferon Regulatory Factor
PAMP: Pathogen-associated molecular pattern
STAT: Signal Transducer and Activator of Transcription Protein
STING: Stimulator of Interferon Genes
TBK1: Tank Binding Kinase 1
Wee1: WEE1 G2 Checkpoint Kinase
