# Supplementary material for: Checkpoint Kinase 1 (Chk1) inhibition fails to activate the Stimulator of Interferon Genes (STING) innate immune signalling in a human coculture cancer system
Source: Mol Biomed. 2021 Jun 20;2:19. doi: 10.1186/s43556-021-00044-1 (PMC8607375; doi:10.1186/s43556-021-00044-1)
Supplement: Supplementary file 1 — Additional file 1: Supplementary Table 1. GI50 and γH2AX EC50 values for V158411. Supplementary Table 2. List of antibodies used. Supplementary Fig. 1. cGAMP but not V158411 increases IFN or NF-kB reporter activation in a time dependent fashion. THP1-Dual cells were treated with 50 μg/mL cGAMP or 3 μM V158411 for 6-72 hours and a reporter activity determined, or b samples prepared for western blotting. Mean of 3 independent wells ± SD. Supplementary Fig. 2. V158411 induces γH2AX in HCC1937 or HT29 cells. a HCC1937 or b HT29 cells were treated with 3x GI50 of V158411 for 24 or 48 hours. After the THP1-Dual reporter activity was determined, the THP1 cells were removed and the HCC1937 or HT29 cells formaldehyde fixed and γH2AX expression determined by high content imaging. Mean of 3 independent determinations ± SD. [file 43556_2021_44_MOESM1_ESM.docx]

**Checkpoint Kinase 1 (Chk1) Inhibition Fails to Activate the Stimulator of Interferon Genes (STING) Innate Immune Signalling in a Human Coculture Cancer System**

Molecular Biomedicine

Teresa Brooks, Joanne Wayne and Andrew J. Massey*

Vernalis (R&D) Ltd, Granta Park, Abington, Cambridge, CB21 6GB, UK

* Corresponding Author

E-mail: a.massey@vernalis.com

ORCID Andrew J. Massey: 0000-0002-0276-2573

**Supplementary Table 1.** GI_50_ and γH2AX EC_50_ values for V158411

| **Cell Line** | **GI_50_ (μM)** | **γH2AX EC_50_ (μM)** |
| --- | --- | --- |
| HT29 | 0.65 | 0.80 |
| HCC1937 | 0.36 | 0.26 |
| U2OS | 0.82 | 0.64 |
| THP1 | 0.22 |  |
| Jurkat | 0.50 |  |

**Supplementary Table 2.** List of antibodies used.

| **Target** | **Supplier** | **Prod. Code** | **Spp.** | **Appl.** | **Dil.** |
| --- | --- | --- | --- | --- | --- |
| cGAS | CST | 15102 | R | WB | 1:2500 |
| CXCL10 | CST | 14969 | R | WB | 1:1000 |
| dsDNA | SantCruz | sc57849 | M | IF | 1:1000 |
|  | Abcam | ab27156 | M | IF | 1:1000 |
| GAPDH | CST | 2118 | R | WB | 1:10000 |
| IRF3 | CST | 11904 | R | WB | 1:5000 |
| IRF7 | CST | 4920 | R | WB | 1:1000 |
| NF-κB (p65) | CST | 8242 | R | WB | 1:5000 |
| PD-L1 | CST | 13684 | R | WB | 1:2500 |
| pIRF3 (S396) | CST | 29047 | R | WB | 1:1000 |
| pIRF7 (S477) | CST | 12390 | R | WB | 1:1000 |
| pNF-κB (p65) (S536) | CST | 3033 | R | WB | 1:2500 |
| pSTAT1 | CST | 7649 | R | WB | 1:1000 |
| pSTAT3 (Y705) | CST | 9145 | R | WB | 1:2500 |
| pTBK1 (S172) | CST | 5483 | R | WB | 1:1000 |
| STAT1 | CST | 9172 | R | WB | 1:1000 |
| STAT3 | CST | 9132 | R | WB | 1:2000 |
| STING | CST | 13647 | R | WB | 1:2000 |
| TBK1 | CST | 3504 | R | WB | 1:2000 |
| TREX1 | CST | 12215 | R | WB | 1:2000 |
| γH2AX | Millipore | JBW-301 | M | IF | 1:1000 |
|  | CST | 9718 | R | WB | 1:5000 |

ID., identifier; Appl., application; WB, western blot; IF, immunofluorescence; Spp., species (M, mouse; R, rabbit); Dil., dilution; CST, Cell Signaling Technology


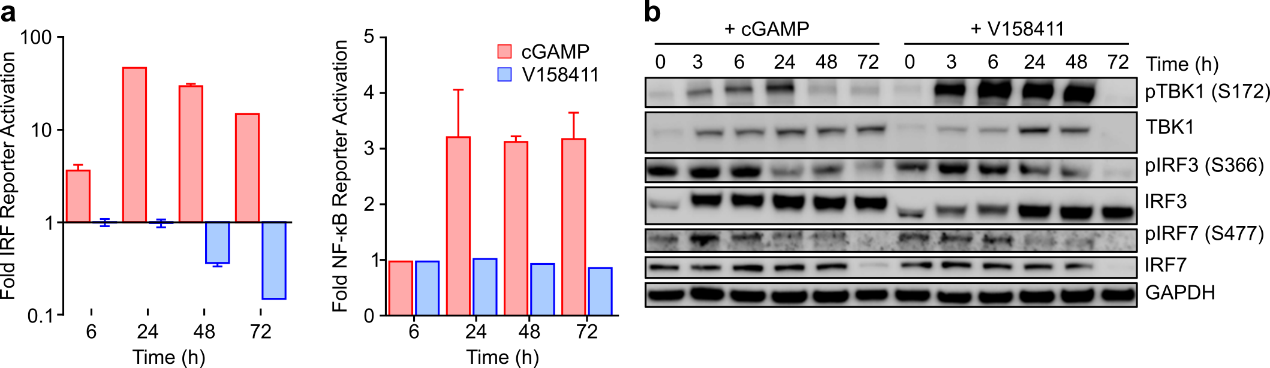


**Supplementary Fig. 1.** cGAMP but not V158411 increases IFN or NF-kB reporter activation in a time dependent fashion.

THP1-Dual cells were treated with 50 μg/mL cGAMP or 3 µM V158411 for 6-72 hours and **a** reporter activity determined, or **b** samples prepared for western blotting. Mean of 3 independent wells ± SD.


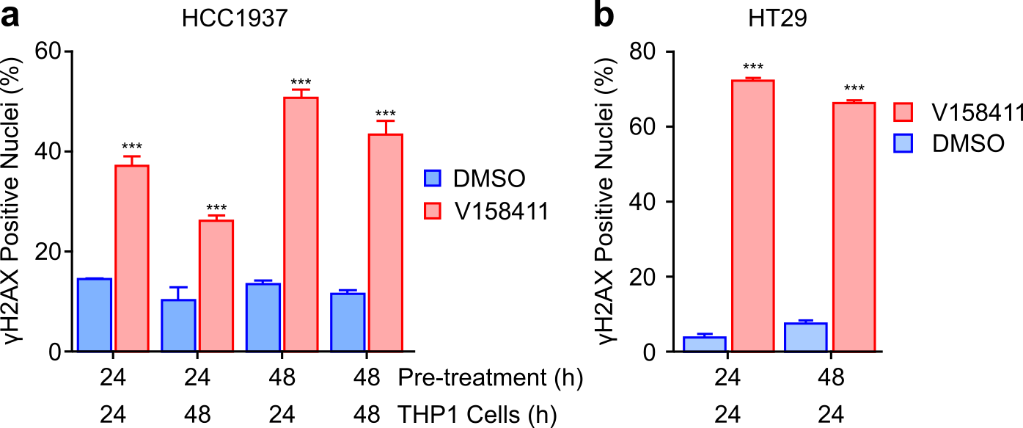


**Supplementary Fig. 2.** V158411 induces γH2AX in HCC1937 or HT29 cells.

**a** HCC1937 or **b** HT29 cells were treated with 3x GI_50_ of V158411 for 24 or 48 hours. After the THP1-Dual reporter activity was determined, the THP1 cells were removed and the HCC1937 or HT29 cells formaldehyde fixed and γH2AX expression determined by high content imaging. Mean of 3 independent determinations ± SD.
